# Case Report and literature review: Delayed diagnosis of ARCL1B due to a newly reported homozygous mutation c.464A>C p. (Tyr155Ser) in the *EFEMP2* gene

**DOI:** 10.3389/fgene.2024.1453195

**Published:** 2024-12-23

**Authors:** Lixue Ouyang, Fan Yang, Hongyu Duan, Chuan Wang

**Affiliations:** ^1^ Department of Pediatrics, West China Second University Hospital, Sichuan University, Chengdu, Sichuan, China; ^2^ Key Laboratory of Birth Defects and Related Diseases of Women and Children, Ministry of Education, Chengdu, China; ^3^ Key Laboratory of Development and Diseases of Women and Children of Sichuan Province, West China Second University Hospital, Sichuan University, Chengdu, Sichuan, China

**Keywords:** autosomal recessive cutis laxa type 1B, ARCL1B, EFEMP2, heart failure, arterial dysplasia

## Abstract

**Background:**

Autosomal recessive cutis laxa type 1B (ARCL1B) is an extremely rare disease characterized by severe systemic connective tissue abnormalities, including cutis laxa, aneurysm and fragility of blood vessels, birth fractures and emphysema. The severity of this disease ranges from perinatal death to manifestations compatible with survival. To date, no cases have been reported in the Chinese population. Due to its rarity, the disease is susceptible to misdiagnosis or missed diagnosis by clinicians. By presenting this case and reviewing the relevant literature, the aim is to enhance clinicians’ awareness and vigilance in diagnosing this disease.

**Case presentation:**

We report a 7-month-old Chinese male infant who initially presented with severe respiratory infection, respiratory failure, and heart failure, and was misdiagnosed with Takayasu arteritis. Despite treatment, his condition did not improve. Due to the features of vascular malformations, developmental delay, and early onset of the disease, whole exome sequencing (WES) was performed, results revealed a homozygous mutation c.464A>C in exon 5 on the *EFEMP2* gene p. (Tyr155Ser) that had never been reported before. Molecular protein prediction results suggest that this mutation site exhibits a high probability of pathogenicity. Combining the clinical manifestations, the results of cardiac color ultrasound and cardiac great vessels angiography, and the WES results, the patient was finally diagnosed with ARCL1B. Given the absence of established guidelines for the clinical manifestation, treatment, follow-up, and prognosis of ARCL1B, we searched the literatures of pubmed and web of science from inception to February 2024 to provide an essential reference for physicians to deepen the understanding of ARCL1B.

**Conclusion:**

The *EFEMP2* gene mutation identified in this patient has not been previously reported, expanding the mutation spectrum of the gene. This is the first documented case of this disease in the Chinese population. The diagnostic and therapeutic journey of this patient, along with the accompanying literature review, provides valuable insights. It highlights the importance of clinicians maintaining a high level of vigilance when encountering cases involving younger patients with multiple pulmonary artery aneurysms, as they may indicate the presence of this rare disease.

## Introduction

Autosomal recessive cutis laxa type 1B (ARCL1B) is a rare disease manifested with severe systemic connective tissue abnormalities, involving the skin, cardiovascular structures, skeleton, as well as the pulmonary system (Hoyer et al., 2009). The cause of ARCL1B is homozygous or compound heterozygous mutation in the *EFEMP2* gene (alias *FBLN4*, OMIM 604633), located on chromosome 11q13.1, which encodes the EGF-containing fibulin-like extracellular matrix protein 2 (also named fibulin-4 protein) and is required for the formation, binding, and functioning of mature elastic fibrils. This fibulin-4 protein, which is involved in the elastic fiber assembly and is related to smooth muscle cell differentiation as well as aortic contractility, is found in the middle layer of the great arteries and veins (Huang et al., 2010; Berk et al., 2012; Hibino et al., 2018). Previous animal studies have proved that reduced expression of fibulin-4 leads to aneurysm formation, dissection of the aortic wall, and cardiac abnormalities (Hanada et al., 2007).

Due to the rarity of the disease and the atypical clinical manifestations in some cases, misdiagnosis and/or delayed diagnosis of ARCL1B is quite easy and common in clinics, which might lead to fatal outcomes due to the high incidence of arterial dysplasia, intractable cardiorespiratory failure and shock (Kappanayil et al., 2012). Therefore, it is urgent to improve the awareness and recognition of clinicians for this rare disorder. To our knowledge, less than 20 literatures of ARCL1B have been currently reported and no cases have been documented in the Chinese population.

Herein, we firstly reported an ARCL1B case with severe respiratory and heart failure as the primary manifestation in the Chinese population, who was initially misdiagnosed as Takayasu’s arteritis and finally confirmed by genetic test revealing a homozygous mutation of c.464A>C p. (Tyr155Ser) in exon 5 of the *EFEMP2* gene, which had never been reported before. Additionally, all the ARCL1B cases with *EFEMP2* gene mutation reported before were reviewed, aiming to provide a reference for the diagnosis, treatment and follow-up of this rare disease and improve the overall prognosis.

## Case presentation

A seven-month-old boy was hospitalized in the cardiovascular intensive care unit of our hospital due to fever, cough with white frothy, cyanosis of the lips, shortness of breath, and languidness for 2 weeks. His family has been living in the Tibet Autonomous Region of China for an extended period, and his parents denied consanguineous. He has no siblings and denied the presence of hereditary diseases within the family. On arrival, the physical examination were as follows: temperature 37.4°C, heart rate 144 beats/min, respiratory rates 62 times/min, oxygen saturation 90% (without oxygen inhalation), blood pressure was measured as 77/50 mmHg in the right upper limb, 94/59 mmHg in the right lower limb, 81/42 mmHg in the left upper limb, and 86/53 mmHg in the left lower limb; edema of eyelids and lower limbs, dyspnea with three depression sign, a large amount of moist rale could be heard in both lungs, the cardiac boundary was enlarged, a grade 2/6 systolic murmur could be heard between the third and fourth ribs of the left sternum, and the liver was enlarged to 2.5 cm below the ribs. He now weighs 6.3 kg, smaller than -2SD of the same age in Chinese boys (Capital Institute of Pediatrics et al., 2005). He is unsteady on his head, unable to roll over and sit alone, indicating obvious retardation of growth and neuromotor.

The ROSS heart failure score of this infant was 7 points, including less than 60 mL of milk per feeding (2 points), feeding time greater than 40 min (1 point), breathing more than 60 times per minute (2 points), abnormal breathing pattern (1 point), and 2.5 cm below the costal margin of the liver (1 point). Combined with the symptoms, signs and the ROSS score of the infant, he was diagnosed with heart failure and was arranged laboratory and imaging tests to uncover the underlying etiologies for heart failure.

**FIGURE 1:**
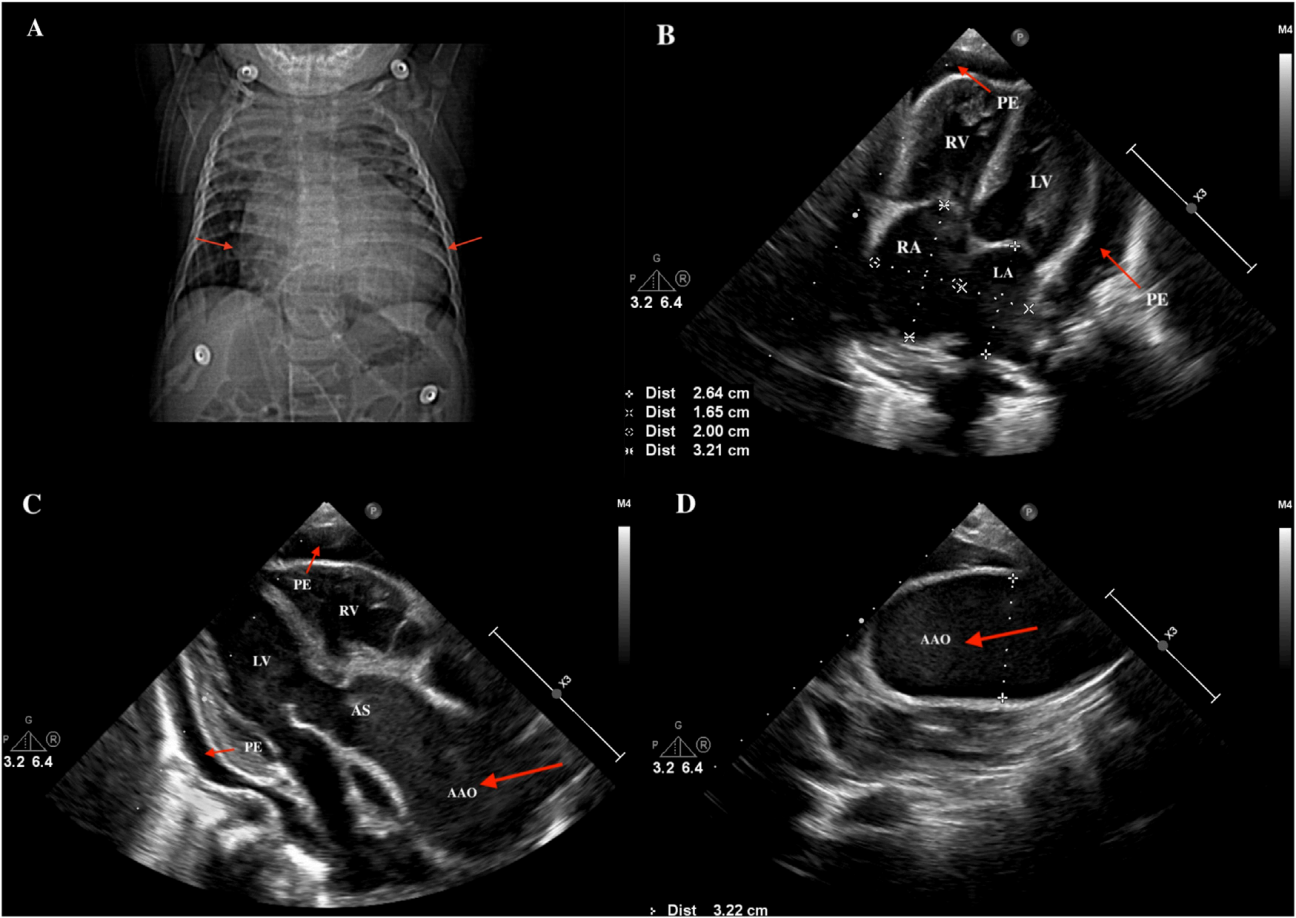
The Results of chest X-ray and cardiac color ultrasound. **(A)** Chest X-ray showed an enlarged cardiac boundary (red arrow). **(B)** cardiac color ultrasound indicated enlargement of the left and right atria (RA 20*32mm, LA 17 mm) and pericardial effusion (red arrow). **(C)** Ascending aortic aneurysmal dilation and pericardial effusion (red arrow). **(D)** Aneurysmal dilated ascending aorta (red arrow). PE, pericardial effusion; RV, right ventricle; LV, left ventricle; RA, right atrium; LA, left atrium; AS, aortic sinus; AAO, ascending aorta.

The result of C-reactive protein (CRP) was 19.2 mg/L (reference value: 0–8 mg/L). Chest X-ray showed an enlarged cardiac boundary (Figure 1A). Color ultrasonography (Figures 1B–D) of the heart indicated enlargement of the right atria (RA 20*32 mm, Z = 4.25; LA 17mm, Z = 0.16), as well as the ascending aorta (24mm, Z = 9.43). The internal diameter of the transverse aortic arch was 32mm, and a distorted, tortuous branch artery was found. The pulmonary artery was widened (MPA 15mm, Z = 3), and its branches crossed and were tortuous. Pericardial effusion (4.0mm–10.0 mm) was also found. No specific signs were found for electrocardiogram (ECG).

**FIGURE 2:**
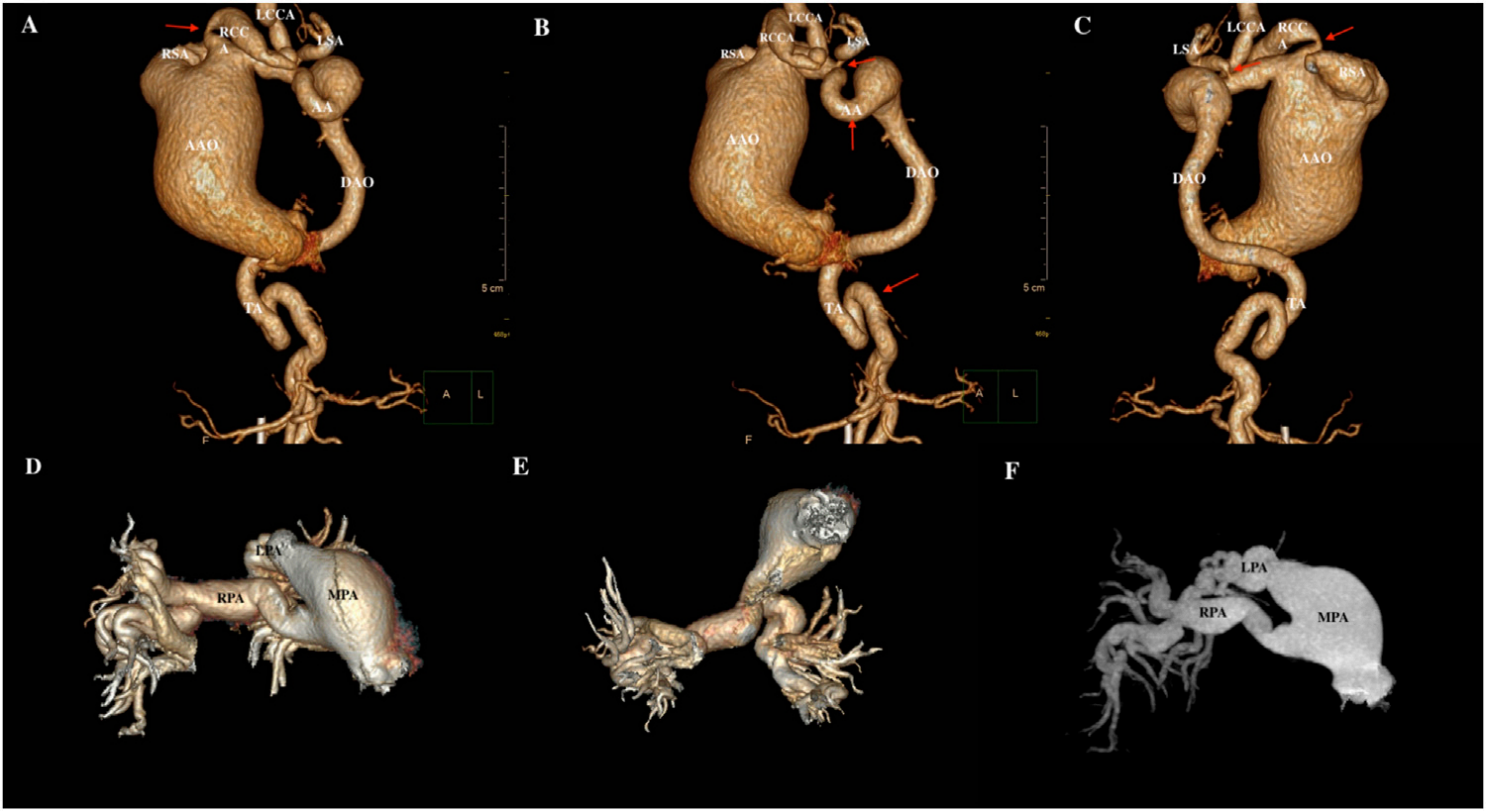
The CTA results of cardiac and great vessels. **(A–C)** The ascending aorta was severely dilated with branch tortuosity and stenosis (red arrow), and arcus transverse aorta tortuosity (red arrow) with aneurysmal dilatation. **(D–F),** Dilated, curved main pulmonary artery and its branches. RSA, right subclavian artery; RCCA, right common carotid artery; LCCA, left common carotid artery; LSA, left subclavian artery; AA, aortic arch; AAO, ascending aorta; DAO, descending aorta; TA, thoracic aorta; MPA, main pulmonary artery; RPA, right pulmonary artery; LPA, left pulmonary artery.

Given the findings of echocardiography, chest vascular computerized tomography angiography (CTA) was further performed. As shown in Figure 2, it showed bilateral pulmonary inflammation with local lung consolidation and emphysema, bilateral local pleural thickening; ascending aortic aneurysmal dilatation, truncus brachiocephalic, right common carotid artery, right subclavian artery, left common carotid artery, left subclavian artery, aortic arch, and thoracic aorta showed obvious tortuosity, with uneven lumen thickness and formation of multiple collateral circulations at both cervical roots. The right atrium and ventricle were enlarged, the right ventricular wall was thickened, and the inferior vena cava and hepatic veins were dilated. The pulmonary trunk thickened, the left and right pulmonary trunk and its branches were obviously tortuous, the lumen thickness was uneven, and the distal bifurcations of the left and right pulmonary arteries were irregular.

Despite the younger age and absence of hypertension, Takayasu’s arteritis was initially considered as the protopathy due to the fever, severe tortuosity and stenosis of multiple major arteries and elevated CRP. Considering the diagnosis was not particularly clear, the patient was only administered oral prednisone at a dosage of 2 mg/kg/day, and immunosuppressants such as methylprednisolone were not given.

However, the treatment didn’t go well, the heart failure went back and forth. In the course of continuing anti-heart failure treatment, we noticed that the patient’s skin was slightly relaxed, but not obviously, which we previously considered to be due to malnutrition. Whereafter, we reviewed the patient’s condition and found that the onset of age is pretty young, and he also had normal blood pressure, and the form of vascular involvement can’t exclude congenital dysplasia of connective tissue such as Loeys-Dietz syndrome, arterial tortuosity syndrome, and Marfan syndrome. Then, the whole exome sequencing (WES) was conducted for this child. However, his parents refused to draw blood for WES due to religious reasons.

**FIGURE 3:**
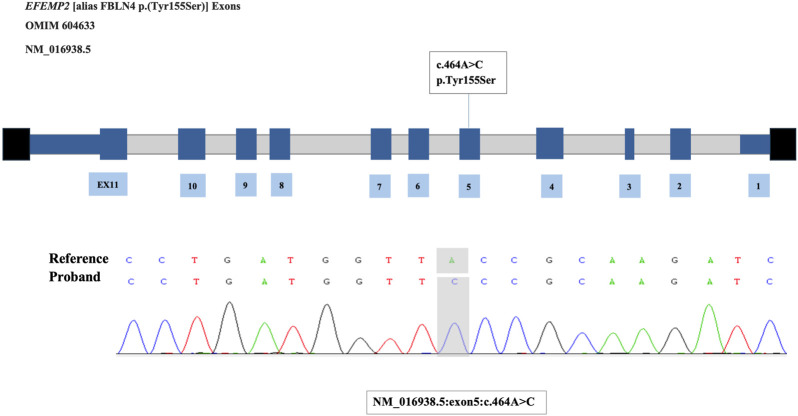
The mutation of EFEMP2 gene and Sanger sequencing verification.

Surprisingly, the results of WES revealed there was a homozygous mutation of c.464A>C p. (Tyr155Ser) in exon 5 of the *EFEMP2* gene in this patient that had never been reported before and verified by Sanger sequencing (Figure 3). Combined with the mild skin relaxation, multiple pulmonary and aortic abnormalities, and genetic results, the patient was finally diagnosed with autosomal recessive cutis laxa type 1B (ARCL1B). We reviewed all the literatures on this disease, which indicated that there is no specific drug for treatment. Many studies reported that some patients underwent surgical treatment. Therefore, we consulted with vascular surgery. Considering that the patient’s condition has not deteriorated further, it was suggested to opt for conservative treatment for now, with follow-up scheduled for later. We continued to provide non-invasive ventilator support. Additionally, digoxin at a dosage of 4ug/kg/dose twice daily, captopril at a dosage of 0.3 mg/kg/dose every 8 h, and hydrochlorothiazide and spironolactone, both at a dosage of 1 mg/kg/dose twice daily, have been administered for treatment. After 42 days of treatment, the patient has recovered and been discharged from the hospital. After discharge, the patient had a routine outpatient follow-up after 1 month with no fever, cough, or decrease in activity tolerance. Three months later, the follow-up echocardiogram showed no significant difference compared to the previous result and the patients showed no specific symptoms.

## Further molecular results

**FIGURE 4:**
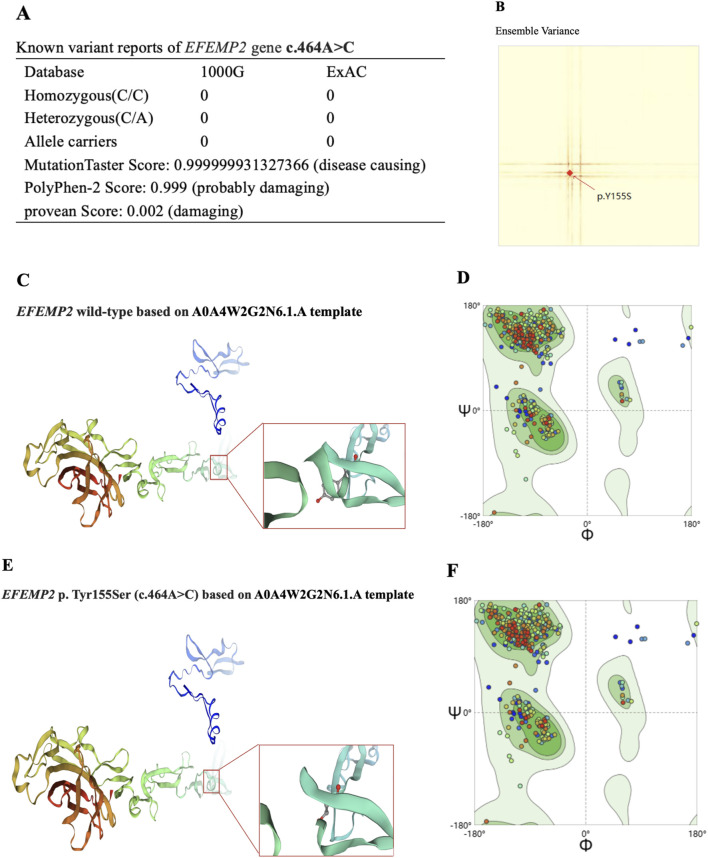
Effects of EFEMP2 c.464A>C mutation on molecular protein structure. **(A)** Prediction of pathogenicity of EFEMP2 wild type and its mutation C.464A>C. **(B)** Ensemble variance of wild type and mutant protein structure. **(C)** Swiss-model of wild-type by using A0A4W2G2N6.1. A template, the protein structure change model. **(D)** Based on **(C)** structural display of Y at place 155. **(E)** Swiss-model of mutational EFEMP2 protein by using A0A4W2G2N6.1. A template, the protein structure change model. **(F)** Based on **(E)** structural display of S at place 155.

Whole-exon sequencing was performed using the BGI DNBSEQ-T7 platform, and a homozygous mutation of c.464A>C p. (Tyr155Ser) in exon 5 of the *EFEMP2* gene was identified. According to the American College of Medical Genetics, the variant was classified as a variant of unknown significance (PM2_Supporting + PP3 + PM3_Supporting). The variant we identified, EFEMP2 c.464A>C, had not been reported in any population, and this is the first report of this variant. An analysis performed with MutationTaster revealed that this mutation is considered pathogenic (probability = 0.999999931327366 for c.464A>C). PolyPhen-2 predicted that this mutation of p. Tyr155Ser to be “probably damaging” (score = 0.999), and the Provean predicted this mutation to be “damaging” (score = 0.002) (Figure 4A). Using SWISS-MODEL protein stability prediction tools, ensemble changes among all the coded amino acids exhibited significant variance (Figure 4B). Ramachandran plots indicated that amino acid positions were altered (Figures 4D, F). Swiss-model was used to model wild-type and mutational EFEMP2 protein. Among the 50 templates, template A0A4W2G2N6.1. A with the highest GMQE value was selected for modeling. Structure Assessment was used to map the protein structure of wild and mutant types (Figures 4C, E). Three types of calculation methods all demonstrated significant destabilizing changes (mCSM = −1.685 kcal/mol; DUET = −1.636 kcal/mol; SDM = −1.3 kcal/mol). We used the FGENESH 2.6 Prediction tool to predict the mutant’s exon structure, yielding a score of 164.459802, which matches the original gene structure and indicates identical splicing to the wild-type, Further details can be found in Supplementary Material S1.

## Literature review of ARCL1B

We searched the literatures of pubmed and web of science from inception to Feburay 2024, performed with no language restriction, by the terms “Autosomal recessive cutis laxa type 1B or ARCL1B, *EFEMP2 or* FBLN4, or *fibulin-4”*. The following indicators were evaluated for each literatures: diagnostic age, Pedigree or population, survival or not, mutation site and tpye, protein effect, consanguinity or not, history of growth and development, facial appearance, development of every organ systems, severe manifestations, surgery or not (see all the detail in Table 1).

**TABLE 1:** Summary of general characteristics of all cases with reported EFEMP2 mutations.

Manifestations	1	2 (Thomas et al., 2021)	3 (Yetman et al., 2019)	4 (Sülü et al., 2019)	5 (Letard et al., 2018)	6 (Hibino et al., 2018)	7 (Rajeshkannan et al., 2014)	8 (Hebson et al., 2014)	9 (Sawyer et al., 2013)	10 (Kappanayil et al., 2012)
Age at diagnosis (mons)	7	84	36	36/4/60/5/27	18WG/10WG	4	MA 1.75 (1days–11years)	6	3/29/24w/3days	MA 1.5
Pedigree or population	N	N	N	Y	Y	N	Y (n = 31)	N	Y	Y (n = 22)
Survival	Y	Y	Y	Y/N/Y/Y/Y	N/N (ternimated24/16WG)	Y	27 (87%)died before 3years	Y	Y/N/N/N	N (76.2% MA 4 mons)
Mutation Type	HOM	HOM	HOM	HOM	HOM	CHM	HOM/CHM	HOM	HOM	HOM(n = 21)/CHM(n = 1)
Mutation site (exon)	c.464A>C (5)	c.608A>C (7)	c.409A>T	c.1189G>A (11)	c.639C>A (7)	NA	c.608A>C (7)/c.679C>T	c.376G>A	c.376G>A (5)	c.608A>C (7)
Protein effect	p.Tyr155Ser	p.Asp203Ala	p.Ser137Cys	p.Ala397Thr	p.Cys213*	NA	p.Asp203Ala/p.Arg227Cys	p.Glu126Lys	p.Glu126Lys	p.Asp203Ala/p.Arg227Cys
Consanguinity	N	N	N	Y	N	NA	Y(One of them)/N	Possible	Y	36.4%
IUGR	NA	NA	NA	NA	NA	NA	NA	NA	NA	—
Postnatal growth deficiency	+	NA	NA	NA	NA	NA	NA	NA	NA	NA
Neuromotor retardation	+	NA	NA	NA	NA	NA	NA	+	NA	100%
Facial appearance										
Craniofacial dysmorphisms	—	—	—	−/−/−/−/−	+/−	—	—	—	−/−/−/−	—
Prominent ears or eyes	—	—	—	−/−/+/−/−	−/−	—	—	—	−/−/−/−	43%
Retrognathia	—	—	—	−/−/−/−/−	+/−	—	—	—	−/−/−/−	43%
Hypertelorism	—	—	—	+/+/+/+/+	−/−	—	—	+	−/−/−/−	57%
high-arched palate	—	—	—	+/+/+/+/+	−/−	—	—	+	−/−/−/−	38%
Skin										
Senile appearance	—	—	—	−/−/−/−/−	−/−	—	—	—	−/−/−/−	—
Skin laxity	+—	+—	+—	−/−/−/−/−	+/−	—	—	+	+/+/+/+	52%
Pulmonary artery dysplasia	+	—	—	—	+	—	+	—	+	+
Emphysema	+	—	—	−/−/−/−/−	−/−	—	—	—	−/−/−/−	—
Cardiovascular										
Dilatation of aortic root	+	+	+	+/+/+/+/+	+/−	+	+	+	+/−/−/+	+
Aortic aneurysm	+	+	+	+/+/+/+/+	−/−	+	—	+	−/+/−/+	+
Arterial tortuosity	+	+	—	+/+/−/−/−	+/+	+	+	+	+/+/−/+	+
Pericardial effusion	+	—	—	−/+/−/+/−	−/−	—	—	—	−/−/−/−	—
Aortic insufficiency	—	+	+	−/−/−/+/+	−/−	+	—	—	−/−/−/−	—
Hernia	—	—	—	−/−/−/+/−	+/−	—	—	—	+/+/−/-	19%
Skeletal abnormalities										
Joint laxity	—	—	—	+/+/+/−/-	−/−	—	—	+	−/−/+/+	29%
Fracture	—	—	—	−/−/−/−/−	+/−	—	—	—	−/−/−/−	—
Hip dislocation	—	—	—	−/−/−/−/−	−/−	—	—	—	+/−/+/−	—
Macrocephaly	—	—	—	−/−/−/−/−	−/−	—	—	—	−/−/−/−	—
Scoliosis	—	—	—	−/−/−/−/−	−/−	—	—	—	−/−/−/−	—
Malformations	—	—	—	—	+/+	—	—	—	−/−/+/−	—
Hypotonia	+	—	—	−/−/−/−/−	−/−	—	—	+	−/+/−/-	43%
Specific manifestation										
Corneal clouding	—	—	—	−/−/−/−/−	−/−	—	—	—	−/−/−/−	—
UTA	—	—	—	−/−/−/−/−	+/−	—	—	—	−/−/−/−	—
Others	—	—	—	—	—	—	—	—	BRF/^−/−^/−	—
Severe infection	+	—	—	−/−/−/−/−	−/−	—	—	+	−/+/−/+	+
Heart failure	+	+—	—	−/−/−/−/−	−/−	—	—	—	−/−/−/−	+
Seizures	—	—	—	—	—	—	—	—	—	18%
Surgery	N	Y	Y	Y/N/Y/N/Y	N/N	Y	NA	Y	Y/N/N/Y	N

11 (Iascone et al., 2012)	12 (Erickson et al., 2012)	13 (Al-Hassnan et al., 2012)	14 (Renard et al., 2010)	15 (Hoyer et al., 2009)	16 (Scherrer et al., 2008)	17 (Dasouki et al., 2007)	18 (Hucthagowder et al., 2006)
0	At birth (31^+2^weeks)	3 to 240	240/84/18	At birth (34weeks)	At birth	At birth (40weeks)	At birth (36weeks)
N	twins	Y (n = 9,4family)	Y (n = 17 + 22; patients = 3)	N	Y	N	N
Y	N(Postnatal resuscitation failed)	Y	Y/Y/N	N	Y	N (died 27days)	N
HOM	HOM	HOM	HOM/HOM/CHM	HOM	HET/HOM/HOM	CHM	HOM
chr11:65638826C>T	c.85delG (1)	c.481G>A (4)	c.376G>A/c.1189G>A/c.377A>T	c.800G>A (7)	c. 776A>G	c.835C>T (8)	c.169G>A
p.Glu57Lys	NA	p.E161K	p.Glu126Lys/p.Ala397Thr/p.Glu126Val	Cys267Tyr	Ile259Val	p.Arg279Cys	p.Glu57Lys
Y	NA	Y	N	Y	N	N	N
NA	NA	NA	NA	Overgrowth	+/+/+	NA	NA
NA	NA	NA	NA	NA	+/+/+	NA	NA
NA	NA	NA	NA	NA	+/+/+	NA	NA
Facial appearance							
—	—	—	−/−/−/	—	+/+/+	—	—
—	+	—	−/−/−/	—	−/−/+	—	—
—	—	—	+/−/−	+	−/−/−	—	—
—	+	—	−/+/+	—	−/−/-	—	—
—	—	—	+/+/−	—	−/−/-	—	—
Skin							
—	—	—	−/−/−	—	+/+/+	—	—
+—	+	—	velvety skin/−/−	+	+/+/+	+	+
+	+	—	+	+	—	+	—
—	—	—	−/−/−	+	−/−/−	—	+
Cardiovascular							
+	+	+	+/+/+	—	−/−/−	+	+
+	—	+	+/+/+	—	−/−/−	+	+
+	+	—	+/+/+	+	−/−/-	+	+
—	—	—	−/−/−	+	−/−/−	—	—
+	—	—	−/−/−	—	−/−/-	—	—
—	—	—	−/−/−	—	+/−/-	—	+
Skeletal abnormalities							
+	—	—	+/+/−	+	−/+/−	—	+
—	+	—	−/−/−	+	−/−/−	—	+
—	—	—	−/−/−	—	−/+/+	—	—
—	—	—	−/−/−	—	−/−/−	—	—
+	—	—	−/−/−	—	−/−/−	—	—
+	+—	—	−/−/−	+	−/−/−	—	—
+	—	—	−/−/−	—	−/−/−	—	+
Specific manifestation							
—	—	—	−/−/−	—	−/−/−	—	—
—	—	—	−/−/−	—	+/+/−	—	—
—	—	—	−/−/−	—	−/−/−	+^a^	—
—	—	—	−/−/−	—	−/−/−	+	—
—	—	—	−/−/+	—	−/−/−	—	—
—	—	—	−/−/−	—	−/−/−	—	—
N	N	Y (89%)	Y/N/N	N	N	N	Y

Notes: N, no; Y, yes; NA, not available; + indicates positive sign; — indicates negative sign; +— indicates the sign is not obvious.

^a^ Arachnodactyly.

Hom, homozygous; CHM, compound heterozygous mutations; HET, heterozygous; WG, weeks of gestation; MA, median age; BRF, baroreceptor reflex failure; UTA, urinary tract abnormalities.

## Discussion

Here, we report the first case of a novel homozygous mutation in the *EFEMP2* gene in the Chinese population. The diagnostic and therapeutic journey for this patient encountered significant hurdles, primarily attributable to a deficiency in the doctors’ comprehension. This experience underscores valuable lessons for future clinical practice.

Cutis laxa is a range of rare disorders of elastic tissue resulting in loose, redundant, inelastic skin that can easily be pulled away from the underlying tissue but only slowly returns to its original position, and there are autosomal dominant, autosomal recessive, X-linked recessive forms (Berk et al., 2012). Previous studies reported that cardiac manifestations are more prominent in ARCL1B, and skin manifestations are more prominent in autosomal dominant, autosomal recessive type 2 and autosomal recessive type 1A of cutis laxa (Berk et al., 2012; Sülü et al., 2019). Our patient’s presentation is consistent with previous literature, as the skin involvement is not particularly apparent, which may be one of the reasons for misdiagnosis. To our knowledge, less than 20 literatures of ARCL1B have been currently reported and no cases have been documented in the Chinese population before. It may be related to the lack of understanding of this disease among pediatricians in China. So it is urgent to improve the awareness and recognition of this rare disorder among clinicians. The early onset and severe involvement, in conjunction with congenital abnormalities across multiple organ systems and abnormal growth development, suggest genetic disorders. Thus, it is crucial to prioritize timely completion of genetic testing.

We have summarized a total of 17 previously reported literatures, along with our own, totaling 18 literatures of *EFEMP2* gene mutation. Consistent with the known function of fibulin-4, the majority of literature (17 out of 18) has reported abnormalities predominantly affecting the cardiovascular system, with aortic aneurysms, arterial tortuosity, and dilation of the aortic root being the most frequently documented. Additionally, occurrences of aortic insufficiency and pericardial effusion have been noted. In our case, the patient presented with significant pericardial effusion, a manifestation that has been infrequently reported in only three previous cases, potentially attributable to the patient’s severe infection. Furthermore, a relatively high incidence of pulmonary artery dysplasia has been observed, with nine out of the 18 articles specifically delineating pulmonary artery abnormalities. These abnormalities manifest as pulmonary trunk dilation (Sawyer et al., 2013; Iascone et al., 2012), pulmonary aneurysm (Dasouki et al., 2007), stenosis (Kappanayil et al., 2012; Rajeshkannan et al., 2014; Renard et al., 2010; Dasouki et al., 2007), tortuosity (Iascone et al., 2012; Dasouki et al., 2007), elongation (Rajeshkannan et al., 2014; Erickson et al., 2012; Renard et al., 2010), pulmonary artery occlusion (Dasouki et al., 2007), and, notably, pulmonary arteriovenous fistulas, as documented in previous reports (Letard et al., 2018). In terms of facial appearance, hypertelorism has the highest proportion, followed by high-arched palate, retrognathia, prominent ears or eyes, and craniofacial dysmorphisms. In terms of skin manifestations, about half of them have skin laxity, and a small part of them show a senile appearance. Additionally, hypotonia, emphysema, hernia, joint laxity, fractures, and hip dislocation are relatively common, though occurring in less than 50% of cases. Conversely, the incidence of severe infection, heart failure, and seizures appears to be relatively lower. Several rare manifestations of the disease have also been documented. For instance, a 38-year-old male experiencing decreased vision in both eyes, uncorrectable with spectacles, was diagnosed with keratoglobus upon ocular examination, marking the first and only reported case of keratoglobus in ARCL1B (Mauger et al., 2019). Rajapakse et al. reported a case of baroreflex failure syndrome (BFS) in a seven-year-old boy with Fibulin-4 Cutis Laxa, characterized by thunderclap headache, vasospasm, malignant hypertension, and dysautonomia. This represents the inaugural instance of BFS secondary to vascular complications of cutis laxa, with notable alleviation following clonidine treatment (Rajapakse et al., 2014). The management of ARCL1B primarily revolves around symptomatic interventions, notably surgical procedures for patients with aneurysms and clear surgical indications, as there is currently no targeted pharmacological therapy available for this condition. Some experts advocate for the administration of β-blockers and angiotensin receptor blockers in cases involving cardiovascular system complications. Additionally, close monitoring through color ultrasound or CTA is recommended for ongoing assessment and timely intervention (Sülü et al., 2019). Currently, the spectrum of disease severity spans from perinatal demise to manifestations compatible with viable existence, thus resulting in variable mortality rates across diverse studies. It has been hypothesized that the deficiency or paucity of mutant fibulin 4 proteins secretion may significantly influence the disease prognosis. The survival outcomes of all documented cases of this ailment are outlined in Table 1. What remains unequivocal is the notably high mortality rate in the initial disease stages, underscoring the pivotal role of prompt diagnosis, therapeutic intervention, and surgical management in enhancing patient survival.

Molecular protein prediction results suggest that this mutation site exhibits a high probability of pathogenicity, previously unreported. The splicing prediction indicates that the mutation does not affect splicing. Despite its high pathogenic potential, further functional validation is warranted.

In summary, a homozygous mutation of c.464A>C p. (Tyr155Ser) in exon 5 of the *EFEMP2* gene in this patient was detected, which had never been reported before, expanding the mutation spectrum of the *EFEMP2* gene, the function needs to be further verified. This is also the first report of this disease in the Chinese population. The diagnosis and treatment process of this patient can provide some meaningful lessons to improve clinicians’ understanding of ARCL1B, so as to make early diagnosis and treatment of this series of diseases, and ultimately improve the prognosis.

## Data Availability

The original contributions presented in the study are included in the article/Supplementary Material, further inquiries can be directed to the corresponding authors.
