# Corrigendum: Case report and literature review: delayed diagnosis of ARCL1B due to a newly reported homozygous mutation c.464A>C p. (Tyr155Ser) in the EFEMP2 gene

**DOI:** 10.3389/fgene.2025.1589037

**Published:** 2025-03-13

**Authors:** Lixue Ouyang, Fan Yang, Hongyu Duan, Chuan Wang

**Affiliations:** ^1^ Department of Pediatrics, West China Second University Hospital, Chengdu, Sichuan, China; ^2^ Key Laboratory of Birth Defects and Related Diseases of Women and Children, Ministry of Education, Chengdu, China; ^3^ Key Laboratory of Development and Diseases of Women and Children of Sichuan Province, West China Second University Hospital, Sichuan University, Chengdu, Sichuan, China

**Keywords:** autosomal recessive cutis laxa type 1B, ARCL1B, EFEMP2, heart failure, arterial dysplasia

In the published article, there was an error in **Affiliation [1]**. Instead of “Department of Pediatrics, West China Second University Hospital, Chengdu, Sichuan, China,” it should be “Department of Pediatrics, West China Second University Hospital, Sichuan University, Chengdu, Sichuan, China.”

The authors apologize for this error and state that this does not change the scientific conclusions of the article in any way. The original article has been updated.

