# Supplementary material for: Case Report and literature review: Delayed diagnosis of ARCL1B due to a newly reported homozygous mutation c.464A>C p. (Tyr155Ser) in the EFEMP2 gene
Source: Front Genet. 2024 Dec 23;15:1453195. doi: 10.3389/fgene.2024.1453195 (PMC11701062; doi:10.3389/fgene.2024.1453195)
Supplement: Supplementary file 1 [file DataSheet1.pdf]

FGENESH 2.6 Prediction of potential genes in Homo_sapiens genomic DNA
Seq name: EFEMP2.c464A>C
Length of sequence: 6360
Number of predicted genes 1: in +chain 1, in -chain 0.
Number of predicted exons 10: in +chain 10, in -chain 0.
Positions of predicted genes and exons: Variant 1 from 1, Score:164.459802

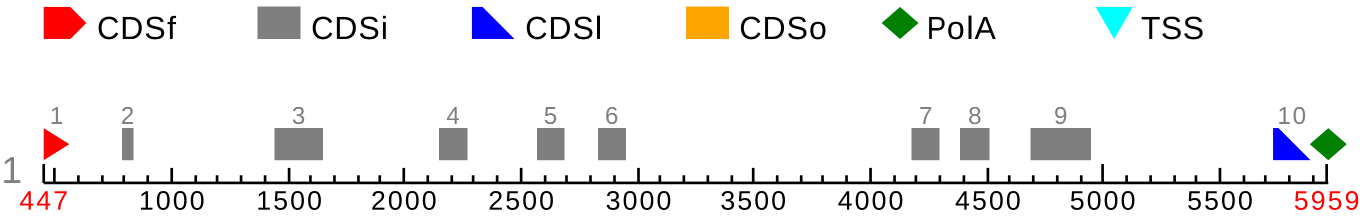

1	+	1	CDSf	447	-	557	15.50	447	-	557	111
1	+	2	CDSi	783	-	831	-0.52	783	-	830	48
1	+	3	CDSi	1438	-	1644	18.93	1440	-	1643	204
1	+	4	CDSi	2143	-	2265	14.08	2145	-	2264	120
1	+	5	CDSi	2564	-	2680	30.20	2566	-	2679	114
1	+	6	CDSi	2825	-	2944	18.96	2827	-	2943	117
1	+	7	CDSi	4172	-	4291	16.17	4174	-	4290	117
1	+	8	CDSi	4380	-	4506	18.63	4382	-	4504	123
1	+	9	CDSi	4682	-	4940	17.96	4683	-	4940	258
1	+	10	CDSl	5722	-	5883	18.65	5722	-	5883	162
1	+		PolA	5959			1.13				

Predicted protein(s):
>FGENESH:[mRNA] 1 10 exon (s) 447 - 5883 1395 bp, chain +
ATGCTCCCCTGCGCCTCCTGCCTACCCGGGTCTCTACTGCTCTGGGCGCTGCTACTGTTG
CTCTTGGGATCAGCTTCTCCTCAGGATTCTGAAGAGCCCGACAGCTACACGGAATGCACA
GATGGCTATGAGTGGGACCCAGACAGCCAGCACTGCCGGGATGTCAACGAGTGTCTGACC
ATCCCTGAGGCCTGCAAGGGGGAAATGAAGTGCATCAACCACTACGGGGGCTACTTGTGC
CTGCCCCGCTCCGCTGCCGTCATCAACGACCTACACGGCGAGGGACCCCCGCCACCAGTG
CCTCCCGCTCAACACCCCAACCCCTGCCCACCAGGCTATGAGCCCGACGATCAGGACAGC
TGTGTGGATGTGGACGAGTGTGCCCAGGCCCTGCACGACTGTCGCCCCAGCCAGGACTGC
CATAACTTGCCTGGCTCCTATCAGTGCACCTGCCCTGATGGTTCCCGCAAGATCGGGCCC
GAGTGTGTGGACATAGACGAGTGCCGCTACCGCTACTGCCAGCACCGCTGCGTGAACCTG
CCTGGCTCCTTCCGCTGCCAGTGCGAGCCGGGCTTCCAGCTGGGGCCTAACAACCGCTCC
TGTGTTGATGTGAACGAGTGTGACATGGGGGCCCCATGCGAGCAGCGCTGCTTCAACTCC
TATGGGACCTTCCTGTGTCGCTGCCACCAGGGCTATGAGCTGCATCGGGATGGCTTCTCC
TGCAGTGATATTGATGAGTGTAGCTACTCCAGCTACCTCTGTCAGTACCGCTGCATCAAC
GAGCCAGGCCGTTTCTCCTGCCACTGCCCACAGGGTTACCAGCTGCTGGCCACACGCCTC
TGCCAAGACATTGATGAGTGTGAGTCTGGTGCGCACCAGTGCTCCGAGGCCCAAACCTGT
GTCAACTTCCATGGGGGCTACCGCTGCGTGGACACCAACCGCTGCGTGGAGCCCTACATC
CAGGTCTCTGAGAAGGTAGGAGAAATTGGGGGGACGGGCAAGGAGCTCATTTGTGTCCCC
CACCTCGGGGTCTCTAGCCGCTGTCTCTGCCCGGCCTCCAACCCTCTATGTCGAGAGCAG
CCTTCATCCATTGTGCACCGCTACATGACCATCACCTCGGAGCGGAGCGTGCCCGCTGAC
GTGTTCCAGATCCAGGCGACCTCCGTCTACCCCGGTGCCTACAATGCCTTTCAGATCCGT
GCTGGAAACTCGCAGGGGGACTTTTACATTAGGCAAATCAACAACGTCAGCGCCATGCTG
GTCCTCGCCCGGCCGGTGACGGGCCCCCGGGAGTACGTGCTGGACCTGGAGATGGTCACC

ATGAATTCCCTCATGAGCTACCGGGCCAGCTCTGTACTGAGGCTCACCGTCTTTGTAGGG
GCCTACACCTTCTGA
>FGENESH: 1 10 exon (s) 447 - 5883 464 aa, chain +
MLPCASCLPGSLLLWALLLLLLGSASPQDSEEPDSYTECTDGYEWDPDSQHCRDVNECLT
IPEACKGEMKCINHYGGYLCLPRSAAVINDLHGEGPPPPVPPAQHPNPCPPGYEPDDQDS
CVDVDECAQALHDCRPSQDCHNLPGSYQCTCPDGSRKIGPECVDIDECRYRYCQHRCVNL
PGSFRCQCEPGFQLGPNNRSCVDVNECDMGAPCEQRCFNSYGTFLCRCHQGYELHRDGFS
CSDIDECSYSSYLCQYRCINEPGRFSCHCPQGYQLLATRLCQDIDECESGAHQCSEAQTC
VNFHGGYRCVDTNRCVEPYIQVSEKVGEIGGTGKELICVPHLGVSSRCLCPASNPLCREQ
PSSIVHRYMTITSERSVPADVFQIQATSVYPGAYNAFQIRAGNSQGDFYIRQINNVSAML
VLARPVTGPREYVLDLEMVTMNSLMSYRASSVLRLTVFVGAYTF
